# Quid Pro Flow

**DOI:** 10.1021/jacs.2c13670

**Published:** 2023-02-14

**Authors:** Andrea Laybourn, Karen Robertson, Anna G. Slater

**Affiliations:** † Faculty of Engineering, University of Nottingham, University Park Campus, Nottingham NG7 2RD, U.K.; ‡ Department of Chemistry and Materials Innovation Factory, University of Liverpool, Crown Street, Liverpool L69 7ZD, U.K.

## Abstract

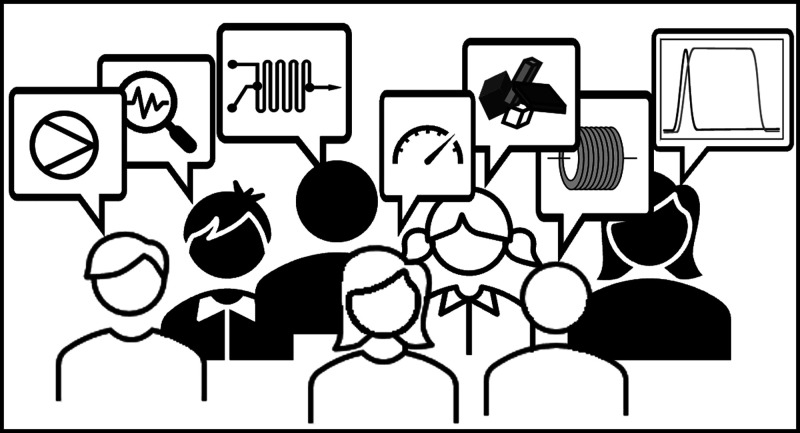

How do you get into flow? We trained in flow chemistry during postdoctoral research and are now applying it in new areas: materials chemistry, crystallization, and supramolecular synthesis. Typically, when researchers think of “flow”, they are considering predominantly liquid-based organic synthesis; application to other disciplines comes with its own challenges. In this Perspective, we highlight why we use and champion flow technologies in our fields, summarize some of the questions we encounter when discussing entry into flow research, and suggest steps to make the transition into the field, emphasizing that communication and collaboration between disciplines is key.

## Introduction

Flow chemistry is a broad, interdisciplinary, and evolving field. The area has its roots in chemical engineering^1,2^ and industrial processes from the beginning of modern chemical production: e.g., blast furnaces, oil refining, and the Haber process. By comparison, flow chemistry in the research lab is a relatively recent development with very different challenges;^3^ new entrants into the field benefit from a long tradition of research,^4,5^ but it can be a challenging or expensive technique to enter into.

Reviews^6−11^ and text books^12−15^ offer invaluable help, especially in fields where extensive examples of the benefits are available: e.g., organic synthesis,^16^ the pharmaceutical industry,^17−19^ and, more recently, nanoparticle synthesis.^20^ Despite this, there are still barriers in terms of skills and knowledge gaps, disciplinary differences, and access to technology that prevent widespread use of flow in chemistry lab settings, particularly in fields where it is not an established technique.

Here, we have addressed those barriers and preconceptions, seeking to demystify the subject and ease the transition to flow. Throughout, we point to resources, literature, and strategies to assist the new user of the technique.

In this Perspective, which does not seek to be exhaustive, we chiefly discuss lab-scale flow processes, with a focus on solution-based chemistry for synthesis and crystallization. We have excluded areas such as continuous mechanochemistry (e.g., twin screw extrusion^21^) and pilot-plant continuous processes, large areas of research in their own right.

## Terminology and First Steps

A shared vocabulary is an important foundation for collaboration between disciplines; conversely, a lack of clear definitions or multiple words being used to mean similar things can be a barrier to entry. There is a myriad of contrasting and nuanced terminology used in *flow chemistry*, in fact, even the field itself is called different things and invokes many disciplines: continuous flow, process engineering, lab-on-a-chip, microfluidics, millifluidics, microreactors, and more. Flow chemistry also takes strong elements from, e.g., reactor design, fluid dynamics, process engineering, etc.; specific terms in use will differ depending on the training and background of the person using them. To alleviate this, there are several reviews aimed at the new flow chemist,^11,22,23^ many of which include explanations of key terminology, e.g., steady state, residence time and residence time distribution, mass and heat transfer, and whether an analytical technique is described as being used offline, in-/online, or at-line. Establishing a shared vocabulary and how terms are used is a sensible starting point at the initiation of a new research partnership.

**Figure 1:**
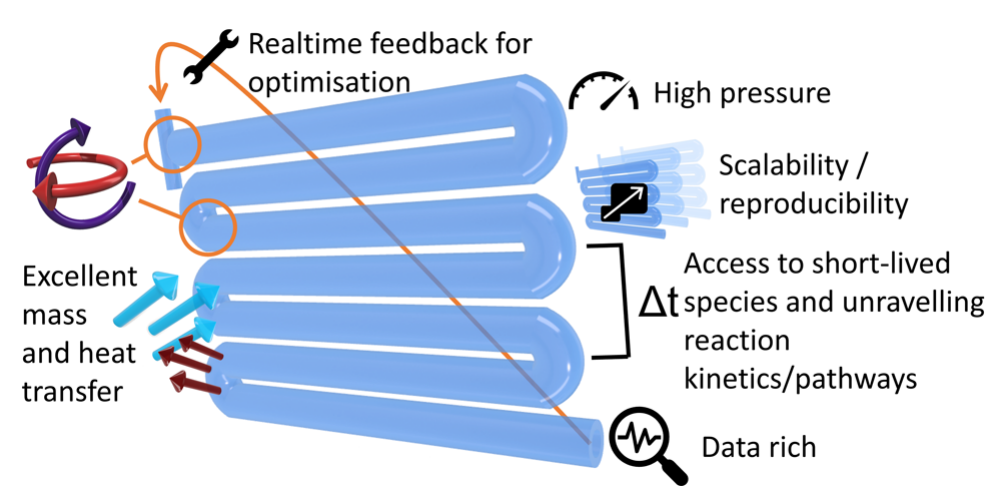
Potential benefits of flow.

The first question is often: “should flow be used at all?” Reviews aimed at answering *when* one should “go with the flow” offer a helpful guide.^24^ Many reactions progress entirely satisfactorily in a round-bottomed flask (RBF); if that is the case, the RBF is the easier solution. However, there are many examples where selectivity, yield, reproducibility, or scale is limited in a batch vessel. Is initial mixing important, or is temperature control critical? Is the reaction time scale too long, or is one of the intermediates highly unstable or hazardous?^25^ Would inline analysis give new information about a process?^26^ In these cases, the challenges in developing flow chemistry skills could well be worth it (Figure 1).

Unlike more common laboratory procedures such as Schlenk line techniques, flow has a somewhat deserved reputation as a technique that takes a long time to perfect. The question here is which skills are required to be a successful flow chemist? It may not be obvious how much engineering knowledge is required to construct and use flow equipment or that a full appreciation of fluid dynamics is not always necessary to interpret the results of an experiment. Manufacturers of commercial equipment have attempted to reduce this skills barrier by developing “plug-and-play” systems that are robust—for example, designed for undergraduate education—but these still require knowledge of steady state, mass and heat transfer, and, of course, reaction design and optimization strategies in the context of flow. However, a brief introduction to these key concepts, undergraduate level physical chemistry, and knowledge of the chemical system under study is enough to start predicting and testing whether flow is likely to bring benefits to a given reaction process.

There is no substitute for learning from people who are experienced in the area, whether through lecture courses, practical training, or research exchanges. Predesigned flow chemistry experiments targeted at undergraduates have been developed and are a useful component of new researchers’ training.^27−29^ However, many users will only start in flow at the postgraduate level: several flow-specific training courses are now available to meet this need. For example, in the UK, the Dial-a-Molecule Network^30^ has offered graduate students and industrial researchers a week-long residential Summer School which includes flow chemistry, reaction design, and 3D printing, among other skills. Likewise, the Flow Chemistry Society, headquartered in Switzerland but active globally, offers events, courses, and textbooks.

As the skills required by graduate chemists change, it is likely that more universities will offer flow chemistry as part of undergraduate lab training, as some centers are already doing. Finally, research visits are a critical part of developing the flow-user ecosystem: all of us benefited from visits to established flow laboratories for proof-of-concept projects, funded by travel and small research grants. Skills in flow chemistry, however, depend on the availability of equipment and tools to apply them; it is more common to see flow equipment in chemistry departments, but this is still far from the standard.

## Equipping the Lab

Once the question of “should” flow be used is answered, and the required training has been acquired, we turn to more practical questions. There is a bewildering array of choices for equipping a flow lab–or even setting up a first system. Navigating these choices is time-consuming and potentially off-putting to the new user.

The first important consideration is the use case: a system primarily designed for education or an introduction to flow will have dissimilar needs than a system for a single class of reaction or one designed to be as flexible as possible. Instructions for building a flow pathway from constituent parts are available^31^ or even the components themselves, such as 3D printed syringe pumps^32^ or microfluidic chips.^33^ In terms of control systems, various approaches have been used to build automatable flow reactors, with authors often publishing code that can be adapted for use in different settings.^34−38^

Again, in the initial stages, dialogue with experts in the field is the best option to avoid a long and potentially expensive period of trial-and-error. Manufacturers of flow equipment are increasingly publishing case studies and application notes which can be extremely helpful to illustrate common problems and strategies to overcome them. There are also several online resources available to aid flow chemists, such as calculators, resources for drawing flow processes,^39^ video demonstrations, and beyond.

Although there are many strategies to transfer a process from batch to flow, depending on the specific challenges of each process, and the eventual solutions may be quite different, there are several common steps that most flow chemists will go through that are a great starting point for each process. Likewise, there are common challenges—such as pump selection and maintenance,^40^ hardware communication, slurry delivery,^41^ sampling frequency, fouling^42^ and blockages, cleaning and troubleshooting^43^—that are part of most flow chemists’ experience, discussed in more detail below.

## Taking a Process into Flow

**Figure 2:**
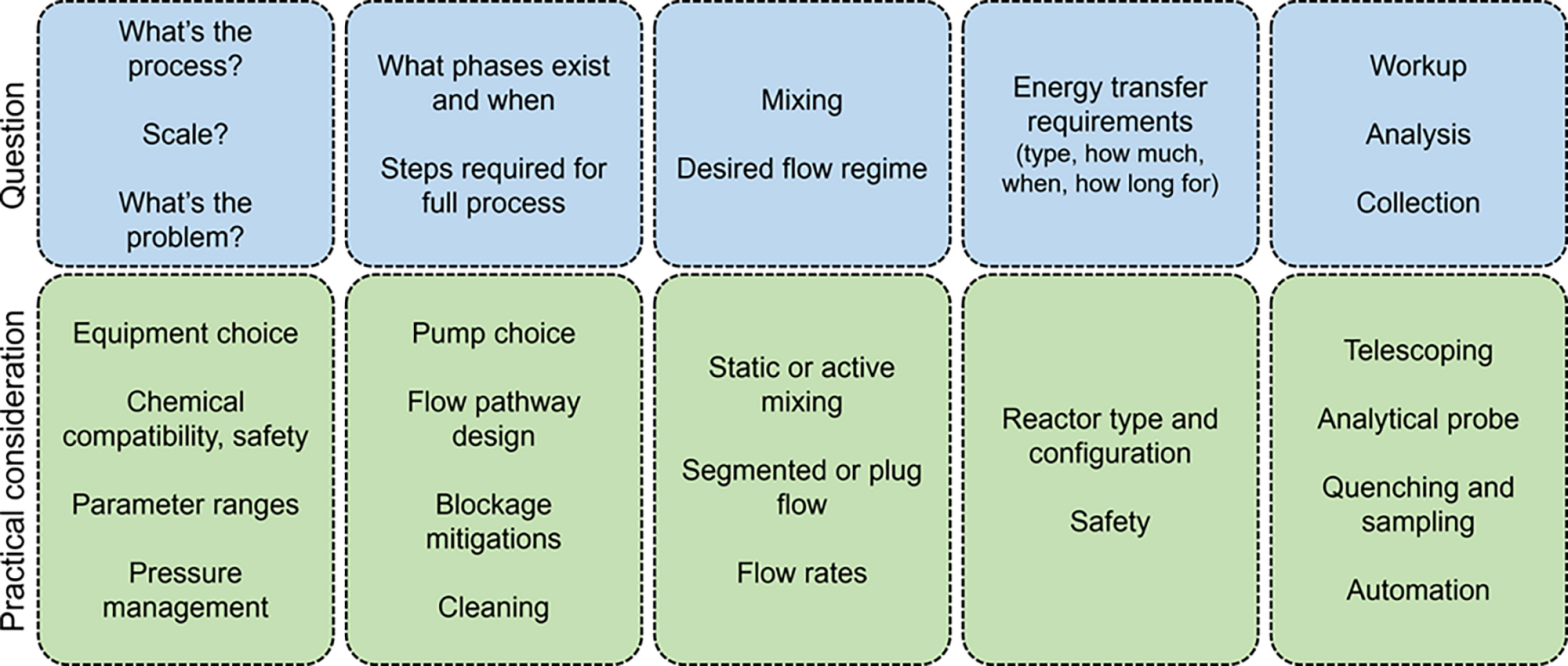
Scheme showing key questions and underpinning practical considerations when taking a process into flow.

A flow chemist with a batch process that they want to improve by translating into flow will consider a series of questions; the order may vary, but common themes will appear (Figure 2). Among the early things to evaluate are the hypothesis to be tested and what the goal is—or conversely, what the issues are with the batch process. For example, does the process involve a hazardous intermediate that needs inline quenching? Does the process scale poorly in batch? Is it highly exothermic and requires careful heat management? Is yield low and likely to be increased by accessing elevated temperatures? Is the selectivity currently limited by the mixing regime accessible in the batch vessel? These all set the scene and allow the flow chemist to start sketching out the equipment and priority considerations for the flow pathway design.

Once the reaction safety, success measures, and, ideally, kinetic information have been understood, we turn to practical considerations. The core steps possible in lab scale flow synthesis are solution delivery, mixing, “reaction”, workup, purification, analysis, and collection.^22^ Several choices underpin these steps, from pump selection, tubing choice, reactor design, and flow pathway planning to telescoping, reaction optimization, and analysis.

It is important to establish the phase or phases present in the system: is the process in the liquid phase and homogeneous throughout, or are solids or gases present or generated at any point? A slurry delivery rules out piston pumps in favor of peristaltic pumps; the production of gases or solvents above their boiling points necessitates the use of back-pressure regulators to maintain steady state conditions and could require a gas/liquid separator in the flow pathway. Equally important for pump choice is the viscosity, chemical compatibility, and hazards of the reagents and required flow rates and scales.

Next, the flow chemist may consider the mixing regime required for the chemistry under study.^44^ Ultimately, the rate limiting step for your process should be identified and characterized in terms of the Damköhler number,^45^ a useful ratio for determining whether the process is diffusion limited. If the process is diffusion limited then high intensity immediate mixing is essential: e.g., micromixers could be used. If the process is not diffusion limited, then simple tee/T-pieces are sufficient. In some cases, it may even be beneficial to have very slow or inefficient mixing or use phase separation to avoid high-dilution and/or effect “slow addition” techniques commonly used, e.g., for macrocycle synthesis.^46,47^

The choice of downstream mixing options depends on whether different viscosities or phases are present, and how best the desired flow rate to mixing ratio can be achieved; as flow rates increase, so too does mixing intensity. Downstream mixing is typically achieved through serpentine bends imparting Dean vortices in microreactors or static mixers, e.g., Kenics or SMX,^48^ in millireactors. Oscillatory flow either through baffles^49^ or combined with static mixing elements^50^ can also help impart high intensity mixing, whereas Taylor or segmented flow generates plug flow (essentially creating microbatch reactors) with varied mixing intensity dependent on tubing/channel size.^51,52^

Next, the question is how to impart the required energy for the process into the system. Many different methods of heating are available to control reaction rate or to enable selective heating: conventional heating baths or blocks now sit alongside photochemical,^53−56^ electrochemical,^57^ microwave,^49,58,59^ or sonochemical^60^ or inductive heat^61^ reactors. Here, the key is ensuring that the technology is appropriately integrated, and all significant parameters are considered: the principles differ based on the type of technology and cannot be generalized. For example, in mechanochemical methods, the geometry of the twin screw extruder is a key consideration, whereas in photocatalysis the reactor should be constructed from materials that allow maximum and uniform light energy transfer. For microwave flow synthesis, key considerations are quantifying the dielectric properties of the reaction mixture and subcomponents with the electromagnetic field (including the reactor itself!) and the electric field distribution as this dictates the power density in the heated phases of the reaction mixture. When working with immobilized reagents such as enzymes,^62^ solid-supported catalysts,^63^ or solid reagents, column packing, pressure drop, the avoidance of “hot zones”, and material surface area are important considerations. Again, collaborating with experts in each technology is recommended to get up to speed quickly.

Wherever possible, the overall efficiency and sustainability of the flow process should be considered from the beginning. In flow, waste minimization is achieved by coupling unit operations, enhanced mass and heat transfer kinetics, and intensified mixing leading to improved process control, conversion efficiencies, and higher yields.^64,65^ Further waste minimization can be accomplished by reusing and recycling, for example, unreacted starting materials and solvent from synthesis and purification steps.^66^ The energy efficiency of a process is enhanced by flow compared to batch through reduced reaction times and operating under steady state conditions. Energy may also be saved by heat recovery, for example, in exothermic reactions.^67^ Alternative sustainable approaches include using “greener” solvents such as supercritical carbon dioxide^68^ or the use of alternative energy sources, as discussed above. Both microwave and photochemical methods offer significantly reduced energy consumption; photochemical methods can use freely available sunlight to power chemical reactions,^69^ whereas microwave technology has the potential to be operated using sustainable electricity obtained from renewable sources including solar, wind, or hydro- and bioenergy. Opportunities also exist for reactions to be driven by electrochemical^57^ and even nonthermal plasma methods.^70−72^

Post-reaction there may be a need for work up steps, especially for telescoped reactions—that is, directly going from one reaction to another within a single flow process. A range of workup steps have evolved to facilitate this including liquid-liquid (or gas-liquid) extraction, inline or online column chromatography,^73,74^ microcrystallization,^75^ microdistillation,^76^ counter-current extraction,^77^ and nanofiltration/dialysis.^78^ Finally, process safety is improved though reduced reactor volumes and control (e.g., relief valves) and monitoring systems and through the ability to produce then consume hazardous intermediates without the need for separation.

Arguably one of the greatest assets of flow chemistry is the ability to have inline and online analysis.^26^ Here, *inline* denotes that the analysis is taken within the flow pathway such that all process fluid passes the analysis point; *online* implies a sample loop such that only a portion of the process fluid is analyzed. Online techniques are typically used if sample prep, such as dilution, is required prior to analysis (e.g., ultra-high performance liquid chromatography, UHPLC, or dynamic light scattering, DLS). By moving the analytical probe or having multiple analysis points, reaction kinetics or pathway can be elucidated, given that flow path length = time under continuous conditions.^79^ As analytical chemistry offers increasingly rapid and sensitive measurements, more techniques are being incorporated into flow processes: LC,^80^ GC,^81,82^ MS,^83,84^ particle sizing,^85^ IR,^86,87^ optical methods,^88^ and NMR^89,90^ are more established, but more recently PXRD,^91,92^ optical emission spectroscopy,^93^ SAXS/WAXS,^94−96^ 3D microscopy,^97^ magnetometry,^98^ and even single crystal XRD^99^ have been reported as inline or online methods. In each case, the method must be optimized for the desired analyte, concentration, and sensitivity required.^100^

At the end of the process, there are collection options to consider: for an accurate comparison of batch vs flow, it is important to quench at the end of the reactor to ensure the reaction is only in progress in the flow pathway and not in the collection vessel. It can be important to confirm that steady state conditions have been reached, particularly if the solution has complex or changing rheological characteristics, and to adjust collection parameters accordingly. Autosamplers can be used as fraction collectors to queue up and collect multiple sequential flow experiments or sample multiple time-points during a single flow experiment and can even be used to integrate with other hardware for automated analysis.

One oft-cited barrier to the use of flow is the handling of solids: this is such a prevalent issue that it merits further discussion in its own section.

## Solids–The Nemesis of the Flow Chemist?

The preconception that “solids mean flow is impossible” may stop such researchers exploring further. It is true that solid handling in flow is a challenge, but there are many engineering solutions that can address this challenge, e.g., slurry handling techniques,^101^ continuous mechanochemistry,^21,102,103^ and continuous stirred tank reactors.^104^ The key question is whether the engineering solution proposed fits the problem in terms of advantages, cost, and the time it takes, the particle size/loading required, and the environmental impact and resources. Often, the simplest strategy is to identify conditions, solvent choices, or temperature or concentration regimes where everything is kept in solution.

If a solid cannot be avoided, or is indeed desired, there are several common strategies to ease the handling of slurries. Such strategies include avoidance of pinch points and narrow tubing, e.g., using peristaltic pumps as back-pressure regulators, avoiding right-angle turns in tubing or connectors, or using wide-bore fittings. Flow pathways can also incorporate measures to homogenize particles, such as inline sonicators^105,106^ or using agitated reactors.^107^ Careful selection of pumps and tubing is required, for example using peristaltic^91^ rather than piston pumps that cannot tolerate particles. Finally, cleaning cycles^108^ can be essential to manage fouling. A combination of all these strategies may be required to arrive at a robust method for a flow process requiring slurry delivery, and it may take time to arrive at a functional solution. However, solid handling poses problems for all chemists, not just those using flow; using flow methods to move a slurry may be an easier and safer option than handling a static, hygroscopic, or highly hazardous powder.

Despite the challenges of solid handling, there are also great advantages in using flow for those deliberately seeking to make bespoke solid particles with a high degree of control. The area of flow crystallization addresses many of these issues with a focus on the control of actively precipitating material.^109^ The use of solids as a slurry which are in steady state—that is, neither crystallizing nor dissolving—can be achieved using the strategies highlighted above. During flow crystallization as an active process, there are greater challenges such as encrustation^110^ (i.e., nucleation and growth of particles on the reactor wall), crystal growth control,^111^ and filtration.^112^

**Figure 3:**
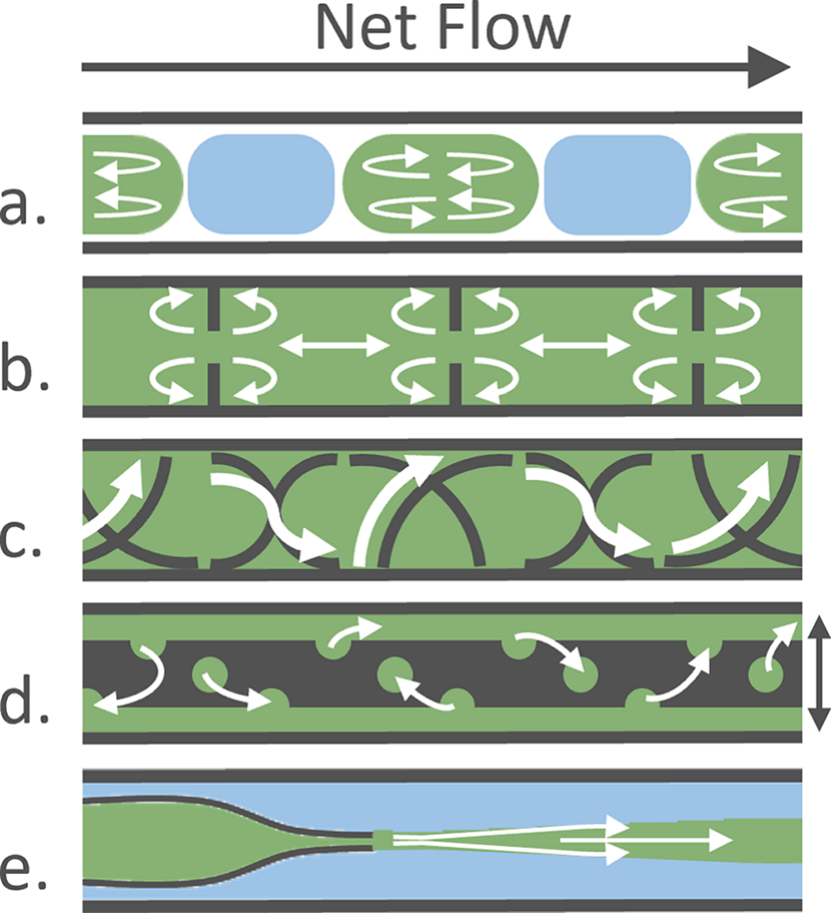
Schematic representation of flow within a range of crystallizers that may be used to improve suspension and mitigate encrustation of particles on reactor walls. White arrows show fluid pathways, solution in green, secondary fluids in blue/white: a. segmented flow, b. continuous oscillatory baffled reactor, c. kenics-type static mixer, d. moving insert (vertical motion of insert denoted by black arrow), e. flow-focused/sheath flow.

Control over how and where material nucleates will prevent unwanted nucleation on the walls of the reactor, which, if unchecked, will lead to fouling, back-mixing, and eventually blockage. The use of antisolvent addition,^113^ rapid cooling,^114^ or sonication^115^ to force material to nucleate can be highly effective. Flow-focusing geometries can direct the location of nucleation away from the reactor walls, further ameliorating encrustation, particularly in microreactors^116,117^ (Figure 3).

Once nucleated, crystal growth can be controlled though temperature gradients^118,119^ or further addition of antisolvent and mixing or suspension of solids.^120^ The most basic downstream mixing setup is a continuous or cascade stirred tank reactor,^104^ which uses a series of stirred tank reactors with an impeller to effect mixing. Plug flow reactors typically employ a variety of static mixers to ensure good mass transfer, reducing agglomeration and improving suspension of solids. Inserts or 3D printed reactors with inbuilt static mixers can help suspend and mix slurries with optimal operation directly linked to flow rate.^121^ Oscillatory baffled reactors (OBRs)^122^ comprise a series of single- or multi-orifice baffles and typically use piston-driven oscillatory flow to generate eddies in the reactor.^123^ A net flow then drives the crystallizing material through the tubing. A combination of oscillation amplitude and frequency and net flow rate provide optimal suspension and plug flow.

Segmented or Taylor flow uses immiscible fluids to separate the crystallizing solution into discrete droplets or “slugs” which mix as a function of flow rate and slug size through bolus flow without contact to a solid surface.^124,125^ Removing contact with the walls inherently prevents encrustation but can raise issues with compatibility of fluids and filtration due to capillary forces of the carrier fluid on the solid particles.

The ideal flow crystallization setup for a given application is a question of particle size, density, and throughput. Nanoparticle crystallization typically requires microreactors to effect the degree of mass transfer control necessary to produce smaller and homogeneous particles.^126^ Due to the small size, blockages with nanoparticles is unlikely but encrustation may be an issue. If particles adhere to the reactor walls, they are likely to grow larger and influence passing growing particles, affecting the particle size distribution. The crystallizer material and surface roughness can therefore be an important decision for all crystallizers, regardless of target size. Traditional flow chip reactors are well-suited to this type of crystallization.^127^ Sub-micrometer sized particles may be more likely to block reactors especially due to agglomeration. Larger, millifluidic reactor parts may therefore be more appropriate, e.g., *ca*. 1 mm internal diameter (ID). These work well with static mixers, small OBRs, and tightly coiled reactor tubing. Milli–macrosized crystallizations, such as small molecule organics, require careful crystallizer planning as the large size makes them very likely to block.^128,129^ Larger static mixer reactors, OBRs, and millisized tubing (*ca*. 3 mm ID) are necessary to prevent blockages with particular care taken for the joints of reactor parts.

Taken together, it is clear that a plethora of engineering solutions have been developed to transfer a process into flow, yet there are still many challenges to be solved.

## Is There Room for Innovation?

Proposing to use flow in research settings sometimes raises a different question: “has this all been done before?” Here, the preconception is that because flow chemistry is firmly established in some areas, there is nothing new to be found.

Indeed, off-the-shelf flow systems and analytical kits are excellent for well-established flow regimes and reaction types; here, the novelty lies in finding new uses for established tools. However, for those focusing on atypical flow (such as venturi mixers, split-recombine or segmented flow) or demanding processes (such as extreme or changing viscosities,^130^ active precipitation of solids, heterogeneous media, or complex temperature gradients)^118,131,132^ these standard set-ups may not be appropriate. Finally, although membrane reactors and packed drying columns can help remove water, there are limited options for a flow equivalent of a Dean–Stark adaptor to help drive condensation reactions.^133^ These challenges present enormous opportunities for research and development, and as new tools are becoming more readily available, it becomes easier to transfer from batch to flow.

Another area of potentially rapid development is the breadth of chemistries that are performed in flow. Traditionally focused on organic synthesis and small molecules, there are now increasing reports of more complex systems being synthesized in flow including supramolecular^46,84,134,135^ and macromolecular materials.^37,90,136−139^ The opportunities that flow offers for enhanced safety and process control also particularly benefit situations where the reagents or transient species are hazardous (e.g., diazonium salts, organometallics, hydrogenation, and fluorination).^25,140−143^

Other major areas of development include automation, real-time inline analysis, and self-optimization, which enables kinetic studies^144^ and rapid screening of reaction and materials space.^145,146^ The potential insight these areas offer for robust discovery and deep understanding of reactions and materials assembly has caught the attention of many newcomers to the field.

Automation in chemistry is a rapidly developing field that is predicted to transform how research is carried out across all areas of science.^147−150^ Flow chemistry is perhaps uniquely suited to benefit from automated methods due to the time resolution and sequential nature of steps in a flow pathway. For example, commercial and custom-build flow platforms are commonly linked with liquid handlers for autosampling^151^ and fraction collection, and pumps are readily controlled by code to automate reaction sequencing, dilution,^152^ screening,^153^ and trigger analysis.^84^ A major selling point of moving to flow is the ability to set up a reaction sequence that screens several parameters while the operator, who can be working entirely remotely,^154^ is freed up to work on other tasks.

A clear benefit of automation is the ability to handle extremely large data sets to rapidly understand and optimize processes. Here, autonomous algorithms are powerful tools to navigate process space and identify ideal conditions in terms of reaction yield, selectivity, throughput, or efficiency.^36,37,145,151,155−163^ “Autonomous reactors” in combination with multifactor optimization have been used to identify flow conditions that are both high yielding and efficient in terms of feedstock use, important in translating lab conditions to process scale.^164^

Optimization algorithms are only effective if robust analytical methods are available for a given system. Here, flow has another advantage: the real-time data that can be collected via inline analysis.^100^ Typically, such measurements are taken under steady state conditions to allow for stable measurement. However, methods have been recently reported that use transient flow measurements without reaching steady state; parameters are continually varied to rapidly explore the impact of, e.g., temperature on kinetics.^165−168^ This powerful technique dramatically shortens the experimental time needed to extract useful information about a process, resulting in many data points from a single run.^90^

Sensitive analytical methods have also enabled extremely high-throughput droplet screening in flow,^169,168−170^ only possible because robust MS data can now be obtained from nanogram quantities. Combining such experiments with autonomous algorithms offers an extremely powerful route to reaction screening and optimization;^171^ here, the backlog becomes data processing and, potentially, generating new hypotheses to test. It will also become increasingly important to train chemists in the skills required for such techniques: coding, data processing, and automation, as well as hardware integration, machine learning approaches, and the limits of these methods.

The increased process windows, reproducibility, and ability to access transient species available in flow coupled with the opportunity for extremely data-rich screening offers a gold-mine of information about the process under study—more than justifying the time and training it takes to develop flow chemistry skills.

## Where Next for Flow?

Alongside the future opportunities discussed, a critical challenge for all chemists is to move away from unsustainable methods of working. Continuous flow reactors can be employed to address principles from process intensification,^172^ green chemistry,^173^ and the circular economy model^174^ such as minimizing waste and/or emissions, energy efficiency, and process safety with the aim of reducing environmental burden. Here, it is important to assess the entire process to quantify the overall environmental impact and to make an informed choice of whether to switch a given process to flow.

There are also enormous opportunities for computational methods to be used at all stages of flow chemistry, from design to discovery to optimization, e.g., predicting complex synthetic targets that are then isolated, optimized, and scaled up in flow. For example, data mining and machine learning have been used to rationalize and accelerate MOF discovery in batch;^175,176^ transferring such approaches to flow would potentially vastly increase the chemical space, data intensity, and scalability of the process. To reach the full potential of flow discovery, there is a need for analytical methods coupled with deep learning algorithms that can interface with harsh and/or complex reaction conditions (particularly multiphase reactions) to analyze and make decisions more rapidly under continuous conditions.

Here, again, the need to collaborate with people across disciplines with differing expertise is apparent: e.g., process chemists, chemical engineers, computer scientists, mathematicians, data scientists, equipment developers and, of course, people with expertise in the types of chemistries being conducted.

## Conclusions

The field of flow chemistry has developed from engineering and retains a strong flavor of industrial research that is perhaps unusual in chemical research communities. Because of that history, chemists may feel barriers to entry in unfamiliar terminology, differing training philosophies, or even how conferences are badged or advertised. However, diversity of mindsets, experiences, and backgrounds is a strength that pushes the field forward, and that relies on welcoming people into the community.

When developers of new technology work in partnership with end users, the feedback and iteration cycle can be extremely rapid. The fast adoption and proliferation of technology can be seen in a number of settings across chemistry and is true for flow too: for example, flow electrochemistry, photochemistry, inline analysis, automation, and autonomous optimization are all developing at a fast pace.

Cross-disciplinary communication is key. We are from very different disciplines and use flow in different ways for different reasons, but the most fruitful discussions come from understanding each other’s perspectives to shed new light on the challenges we face, which are often shared. As more researchers exploit flow, we look forward to seeing the expansion and development of the field into new spaces, and training new generations to benefit from the many opportunities on offer—paying forward the help we have received to join the flow community ourselves and helping to develop flow as a central technique for chemistry.
